# Immunogenic Cell Death Genes Related Prognostic Biomarker in Hepatocellular Carcinoma

**DOI:** 10.32604/or.2025.061422

**Published:** 2025-08-28

**Authors:** Bi Feng, Siqi Yang, Zhiqiang He, Yushi Dai, Ruiqi Zou, Yafei Hu, Haijie Hu, Fuyu Li

**Affiliations:** Division of Biliary Tract Surgery, Department of General Surgery, West China Hospital of Sichuan University, Chengdu, 610041, China

**Keywords:** Immunogenic cell death (ICD), hepatocellular carcinoma (HCC), prognosis, risk score

## Abstract

**Objectives:**

Hepatocellular carcinoma (HCC) is among the most frequently occurring malignant tumors of the digestive tract and is associated with an increased mortality rate worldwide. This study aimed to develop and validate a prognostic model based on immunogenic cell death (ICD)-related genes to predict patient survival and guide individualized treatment strategies for HCC.

**Methods:**

ICD-related genes were identified from the GeneCards database using a relevance score threshold of >10. A combination of least absolute shrinkage and selection operator (LASSO) regression and multivariate Cox analysis was used to screen prognostic genes and construct a risk score model. Immune cell infiltration was evaluated through single-sample gene set enrichment analysis (ssGSEA) and cell-type identification by estimating relative subsets of RNA transcripts (CIBERSORT) algorithms. Associations between risk groups and the tumor microenvironment (TME), N6-methyladenosine (m6A) regulators, and immune checkpoint expression were analyzed. Drug sensitivity was predicted based on the risk stratification. The reliability of the model was validated in internal cohorts and further confirmed by quantitative reverse transcription polymerase chain reaction (qRT-PCR) and immunohistochemistry (IHC).

**Results:**

A six-gene signature (CFHR3, G6PD, IGHM, KPNA2, PON1, and SERPINE1) was identified and used to calculate the risk scores. This study found that high-risk patients exhibited significantly poorer overall survival in both the training and validation datasets. The nomogram integrating the risk score and clinical factors showed strong predictive performance. High-risk patients demonstrated reduced immune cell infiltration, altered expression of immune checkpoints and immunosuppressive factors, and a distinct m6A modification pattern, suggesting a higher likelihood of immune escape. This study also revealed that the risk model effectively predicted sensitivity to multiple anticancer drugs.

**Conclusion:**

This study developed a robust ICD-related six-gene prognostic model for HCC that can accurately stratify patient risk, reflect the tumor immune landscape, and provide guidance for immunotherapy and personalized treatment strategies.

## 1 Introduction

Liver cancer exhibits a high global incidence and ranks as the second leading malignancy worldwide [1]. Hepatocellular carcinoma (HCC), the predominant subtype, represents over 90% of liver cancer cases [2]. Multiple treatment approaches—including surgical resection, TACE, liver transplantation, ablation, and systemic therapies—are used for HCC management across various disease stages. Although progress has been made in diagnosing and treating HCC, its overall prognosis remains unfavorable, with a 5-year recurrence rate of 60%–70% and survival below 20% [3]. Although alpha-fetoprotein (AFP) is commonly employed for detecting and monitoring liver cancer, its diagnostic utility is hindered by limited sensitivity. Emerging biomarkers such as GALAD (Gender, Age, Lens culinaris agglutinin-reactive AFP (AFP-L3), AFP, and Des-gamma-carboxyprothrombin) and circulating tumor DNA (ctDNA) are currently being extensively explored [4,5]. Therefore, the identification of dependable biomarkers and viable therapeutic targets is crucial to enhance the diagnosis and management of HCC.

Immunogenic cell death (ICD) refers to a distinct category of regulated cell death (RCD) that is initiated under conditions of cellular stress, wherein dying cells emit danger-associated signals that stimulate immune activation and promote adaptive immune responses [6]. ICD is marked by the release or increased expression of danger-associated molecular patterns, including adenosine triphosphate (ATP) and calreticulin (CRT), along with inflammatory mediators and cytokines [7]. These molecules recruit antigen-presenting cells (APCs) or their precursors to RCD sites, facilitating interaction with dying cells, and promoting phagocytosis, APC maturation, and T cell recruitment [8–10]. Growing evidence indicates that ICD-related molecules and mechanisms serve as potential prognostic biomarkers in cancer patients [11]. Moreover, therapy-induced ICD can stimulate antitumor immune responses, thereby improving the effectiveness of chemotherapy, radiotherapy, and targeted therapies. [12]. However, the prognostic and therapeutic relevance of ICD in HCC remains insufficiently explored.

In our study, prognostic ICD-related genes were identified through bioinformatics approaches. A multigene prognostic model was devised to enhance the prediction of HCC outcomes. This model may assist in identifying novel therapeutic targets and informing personalized treatment approaches.

## 2 Materials and Methods

### 2.1 Hepatocellular Carcinoma Dataset Acquisition

The study utilized both clinical and RNA-seq data (The Cancer Genome Atlas (TCGA), Genomic Data Commons (GDC), Liver Cancer (LIHC)), which were acquired from the UCSC Xena platform (https://xena.ucsc.edu/, accessed on 18 March 2024) [13]. Clinical variables such as sex, age, tumor grade, survival status, and TNM staging information were included in the clinical dataset. Using the “caret” package (v6.0) in R, the TCGA cohort was evenly split into training and validation sets with a 1:1 allocation ratio. In addition, single-cell RNA sequencing data (GSE242889), including both tumor and matched adjacent normal tissues from five hepatocellular carcinoma patients, were retrieved from the GEO database. To analyze protein-protein interactions, networks were generated using the STRING database (https://string-db.org, accessed on 18 March 2024). Additionally, immunohistochemical expression profiles were obtained from the Human Protein Atlas (https://www.proteinatlas.org/, accessed on 18 March 2024).

### 2.2 Differential Expression of ICD-Related Genes Analysis

Genes associated with ICD were retrieved from the GeneCards database (https://www.genecards.org, accessed on 18 March 2024), with a selection threshold of relevance scores above 10. Differential gene expression in the TCGA dataset was analyzed using the R package limma (version 3.54), with significance thresholds set at a |log2FC| greater than 2 and a false discovery rate (FDR)–adjusted *p*-value below 0.05. The identified DEGs were visualized in a heatmap generated with the *pheatmap* tool (v1.0.12). Furthermore, functional interactions among these genes were investigated by constructing protein-protein interaction (PPI) networks, followed by network visualization using *igraph* (v1.5.1) and *ggraph* (v2.1) in R.

### 2.3 Construction and Validation of the Risk Model

To uncover ICD-related genes with prognostic value in HCC, we initially performed univariate Cox regression analysis, selecting those genes whose expression levels were significantly associated with patient survival (*p* < 0.05). Subsequently, to avoid overfitting, these candidate genes underwent feature selection via LASSO regression using the “glmnet” R package (version 4.1). The most predictive genes were subsequently incorporated into a multivariate Cox proportional hazards model to establish a robust prognostic signature. Based on the multivariate analysis results, each patient’s risk score was calculated using the equation: risk score = $\sum_{i=1}^{n}\;(\text{expression of Gene }i\;*\;\beta i)$, where *n* represents the total number of genes included in the final model, *i* is the index of each gene, and *β*_*i*_ corresponds to its respective coefficient estimated from the multivariate Cox regression, indicating the gene’s relative influence on clinical prognosis. Patients were categorized into high- and low-risk groups according to whether their calculated risk scores exceeded or fell below the median threshold. Principal component analysis (PCA) was employed to illustrate the distributional differences across the different risk groups. Kaplan–Meier survival curves, along with log-rank tests, were used to compare survival outcomes between the different risk groups. Finally, the predictive performance of the risk model was assessed by generating time-dependent receiver operating characteristic (ROC) curves at 1-, 3-, and 5-year time points using the “survival” R package (version 3.5), providing a comprehensive evaluation of the model’s prognostic accuracy across different follow-up periods.

### 2.4 Construction of a Nomogram

To determine independent prognostic indicators affecting survival in HCC patients, we performed a two-stage analysis incorporating univariate followed by multivariate Cox proportional hazards regression models. A prognostic nomogram was developed utilizing the R packages “survival”, “survminer” (v0.4.9), and “rms” (v6.7). The model was constructed using the training cohort and incorporated variables such as sex, age, TNM classification, histological grade, and clinical stage. Model performance was evaluated using Schoenfeld residuals to assess the proportional hazards assumption, and calibration plots were also used to evaluate the predictive accuracy.

### 2.5 Immune-Related Analysis

Immune cell composition was quantified using single-sample gene set enrichment analysis (ssGSEA) and CIBERSORT to estimate relative abundances within the tumor microenvironment. The ‘GSV’ R package (version 1.48.3A) was utilized to calculate enrichment scores across 13 immune-associated signaling pathways. Immune subtypes were classified based on gene expression data using the “ImmuneSubtypeClassifier” package (v1.0). Stromal and immune components within tumor samples were assessed using the ESTIMATE algorithm, which infers non-tumor cell proportions in tumor tissues from transcriptomic profiles. Additionally, the Tumor Immune Dysfunction and Exclusion (TIDE) algorithm was utilized to assess immune evasion potential between groups with high and low ICD scores (http://tide.dfci.harvard.edu/, accessed on 18 March 2024). Additionally, associations between risk scores and immunological features—including immune checkpoints, suppressive cytokines, and m6A RNA methylation—were also investigated.

### 2.6 Calculation of ICD Scores

ICD scores for each sample were calculated using the “AddModuleScore” function in Seurat R package (v5.1.0). This function computes a relative activity score per cell or sample by averaging the expression of the specified gene set and subtracting the combined expression of control gene sets. Default parameters were applied during the calculation.

### 2.7 Drug Sensitivity Analysis

The “pRRophetic” R package (version 0.5.0) was applied to assess how effectively the risk model predicts drug sensitivity across low- and high-risk patient groups. This evaluation relied on data sourced from the Cancer Genome Project (http://www.sanger.ac.uk/genetics/CGP/, accessed on 18 March 2024).

### 2.8 Functional Enrichment Analysis

To explore the biological functions associated with differentially expressed ICD-related genes, enrichment analyses based on Gene Ontology (GO) terms and KEGG pathways were conducted utilizing the R package “clusterProfiler” (version 4.8.2). GSEA was conducted through the GSEA software (v4.0.3) to identify significantly distinct activity in KEGG terms among patients with varying risk scores.

### 2.9 Quantitative Reverse Transcription Polymerase Chain Reaction (qRT-PCR)

To assess the expression of genes within the risk signature, qRT-PCR was conducted on matched tumor and adjacent normal tissues collected from 10 patients who underwent surgery at West China Hospital of Sichuan University. Total RNA was extracted using the RNA-Quick Purification Kit (Yishan Biotechnology, Shanghai, China), and its integrity and purity were confirmed by an A260/A280 ratio ranging from 1.9 to 2.2. Complementary DNA (cDNA) synthesis was performed with the HiScript III All-in-one RT SuperMix kit (Vazyme, Nanjing, China), with reverse transcription at 50°C for 15 min, followed by enzyme inactivation at 85°C for 5 s. The sequences of primers used for the ICD-related prognostic genes are provided in Table A1. The Ethics Committee of West China Hospital granted approval for this study (approval number: 2022 (1901)).

### 2.10 Immunohistochemistry

Tissue microarrays were subjected to immunohistochemical (IHC) analysis. Paraffin was removed using 100% xylene, followed by stepwise rehydration with 95% ethanol. Tissue sections were subjected to antigen retrieval in Tris-EDTA buffer (pH 9.0) at room temperature for 30 min. After that, we incubated with 0.3% hydrogen peroxide for 10 min. Following this step, nonspecific protein interactions were reduced by incubating the samples in 5% bovine serum albumin (BSA; HUABIO, Cambridge, MA, USA) for 1 h. Slides were incubated overnight at 4°C with the primary antibody (HA1106, diluted 1:150; HUABIO). After washing, the secondary antibody (HA1119, 1:200 dilution; HUABIO) was applied for 1 h at 37°C. IGHM expression was then observed under a light microscope at 200× magnification [14].

### 2.11 scRNA-seq Data Processing and Clustering Dimension Reduction

The Seurat (v5.1.0) R package was utilized to integrate and normalize scRNA-seq datasets. Raw expression matrices were filtered based on three criteria: removal of cells expressing fewer than 300 genes, exclusion of those with mitochondrial gene content exceeding 20%, and retention of genes expressed in at least three cells. The top 2000 highly variable genes were identified using the “vst” approach implemented in the FindVariableFeatures function. Dimensionality reduction was then performed via PCA, narrowing the dataset to 22 principal components. Clustering of cells was subsequently conducted using the FindNeighbors and FindClusters functions, with the resolution parameter configured to 0.4.

### 2.12 Statistical Analysis

We conducted statistical analyses using R software (version 4.2.1), considering *p*-values less than 0.05 as statistically significant. To evaluate the prognostic independence of the constructed risk model, both univariate and multivariate Cox proportional hazards analyses were conducted. For each survival metric, *p*-values, hazard ratios, and 95% confidence intervals were provided.

## 3 Results

### 3.1 Identification of the Differentially Expressed Genes and PPI Networks

**Figure 1:**
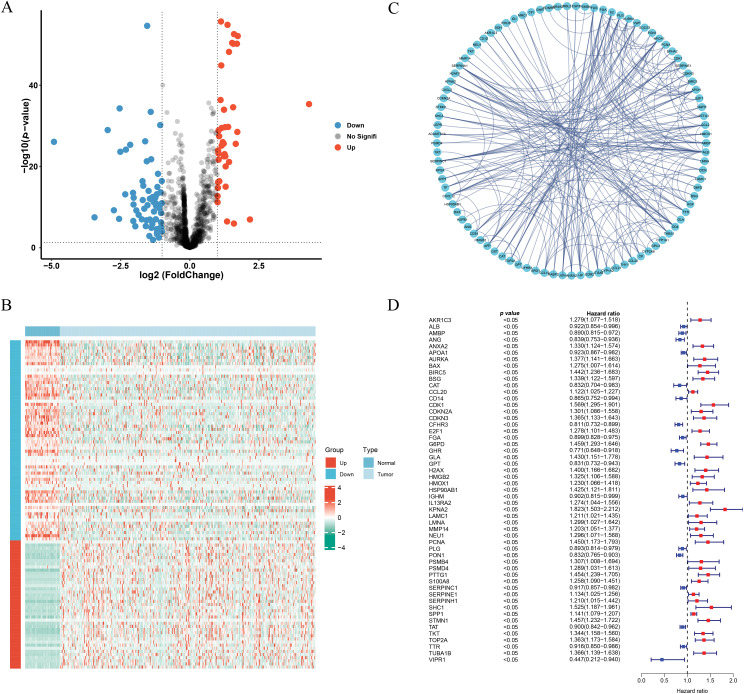
Identification of the ICD-related and prognosis-related DEGs. (**A**) Volcano plot of the ICD-related DEGs. (**B**) Heatmap of the ICD-related DEGs. (**C**) Protein-protein interaction network. (**D**) Univariate cox-regression analysis of ICD-related DEGs

By comparing tumor and normal tissues, 105 differentially expressed genes (DEGs) were identified (Fig. 1A), among which 41 were upregulated and 64 were downregulated in tumor samples (Fig. 1B). The PPI network of these DEGs is illustrated in Fig. 1C.

### 3.2 Risk Score Generation and Verification

**Figure 2:**
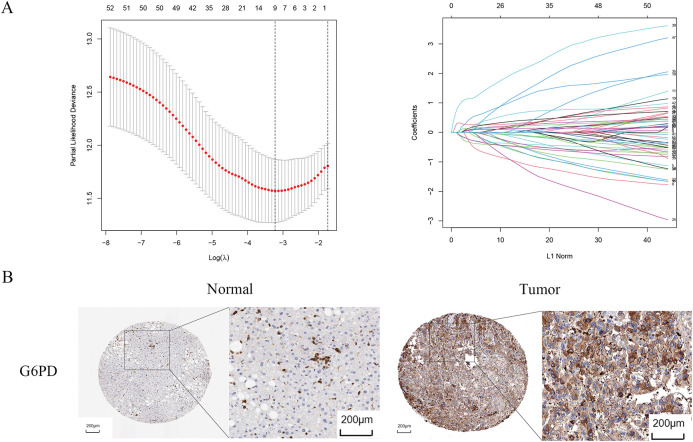

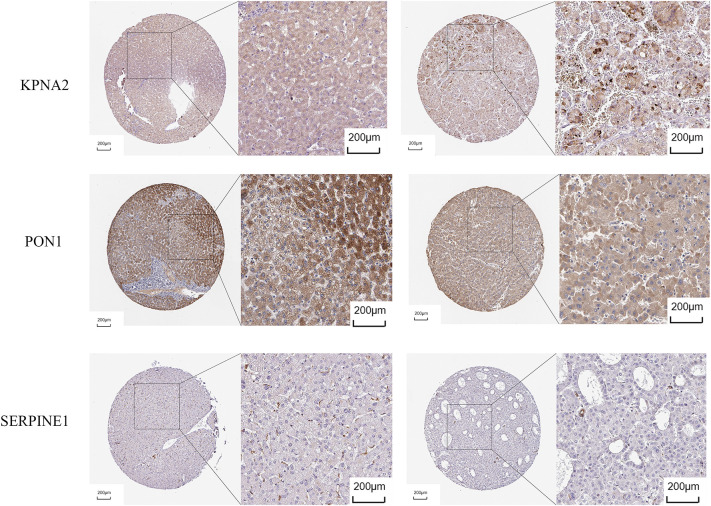
Identification of prognosis-related genes. (**A**) LASSO regression analysis. (**B**) Immunohistochemical images of G6PD, KPNA2, PON1, SERPINE1

**Figure 3:**
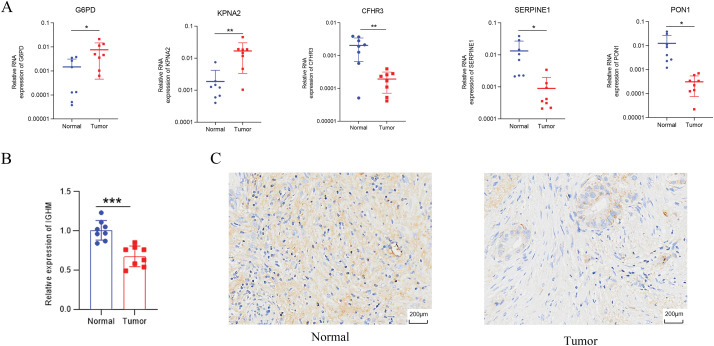
Validation of identified genes. (**A**) G6PD, KPNA2, CFHR3, SERPINE1, and PON1 expression examined by qRT-PCR. (**B**,**C**) Immunohistochemistry of IGHM. *p < 0.05, **p < 0.01, ***p < 0.001

**Figure 4:**
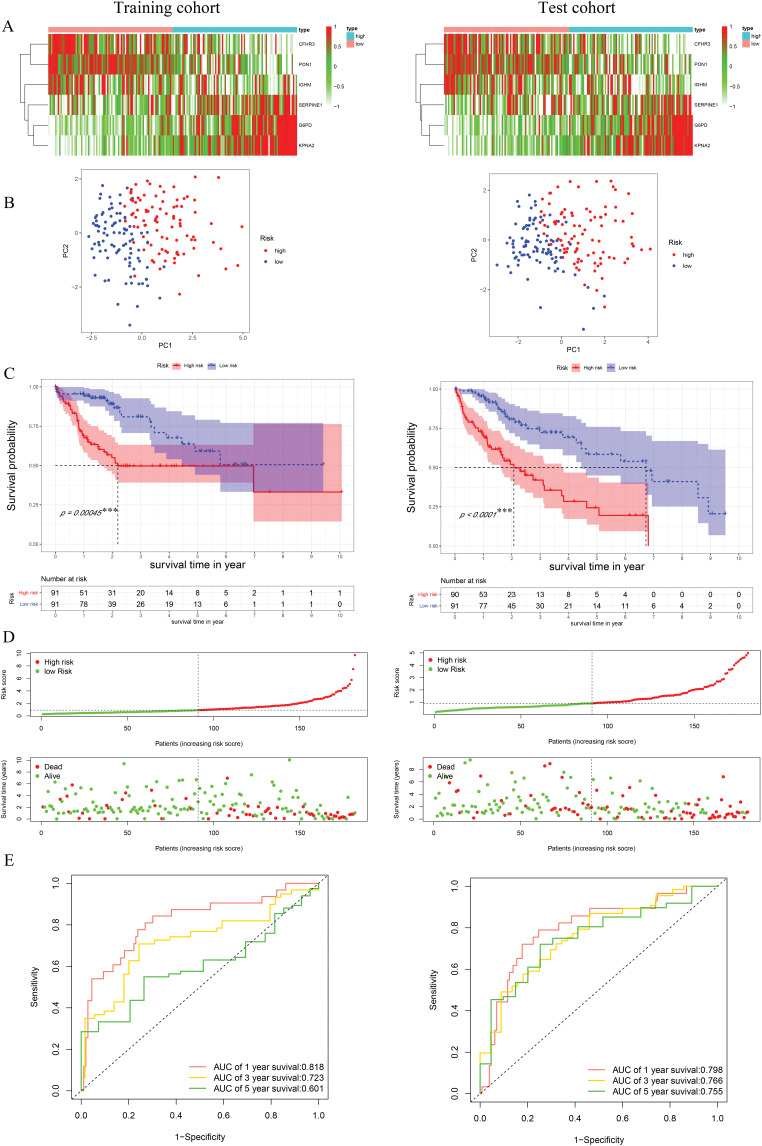
Construction and validation of risk model. (**A**) The heatmap of identified genes in HCC. (**B**) Principal component analysis. (**C**) K-M survival curves of high- and low-risk groups. (**D**) Distribution of the survival, mortality, and risk score. (**E**) ROC curve with the AUCs of 1-, 3- and 5-year survival. ***p < 0.001

Univariate Cox regression identified 53 DEGs associated with HCC prognosis (Fig. 1D). A prognostic risk model was constructed by further narrowing these DEGs using LASSO and multivariate Cox regression analyses (Fig. 2A), ultimately yielding six genes: CFHR3, G6PD, IGHM, KPNA2, PON1, and SERPINE1. Due to the absence of immunohistochemical images for CFHR3 and IGHM, only the remaining four genes were visualized using data from the Human Protein Atlas (Fig. 2B). Expression levels of CFHR3, G6PD, KPNA2, PON1, and SERPINE1 were validated by qRT-PCR (Fig. 3A). As IGHM primers lacked specificity, its expression was assessed by immunohistochemistry (Fig. 3B,C). The experimental results aligned with prior analyses. On the basis of the risk score equation: risk score = (−0.089) × CFHR3 + 0.172 × G6PD + (−0.155) × IGHM + 0.316 × KPNA2 + (−0.090) × PON1 + 0.090 × SERPINE1, individual risk scores were calculated for each patient in both training and test cohorts. Heatmaps revealed consistent expression patterns of the six genes across cohorts (Fig. 4A). Patients were divided into high- and low-risk categories according to their risk scores. Principal component analysis (PCA) illustrated distinct clustering of these two groups across both cohorts (Fig. 4B). Survival analysis using the Kaplan–Meier method showed significantly poorer survival outcomes for individuals in the high-risk category (Fig. 4C), with risk scores negatively correlating with the length of survival (Fig. 4D). Within the training cohort, the time-dependent ROC analysis produced area under the curve (AUC) values of 0.818, 0.723, and 0.601 for forecasting survival at 1, 3, and 5 years, respectively. Corresponding AUCs in the validation dataset were 0.798, 0.766, and 0.755, indicating that the model possesses strong prognostic accuracy (Fig. 4E).

### 3.3 Construction of Predictive Nomogram

**Figure 5:**
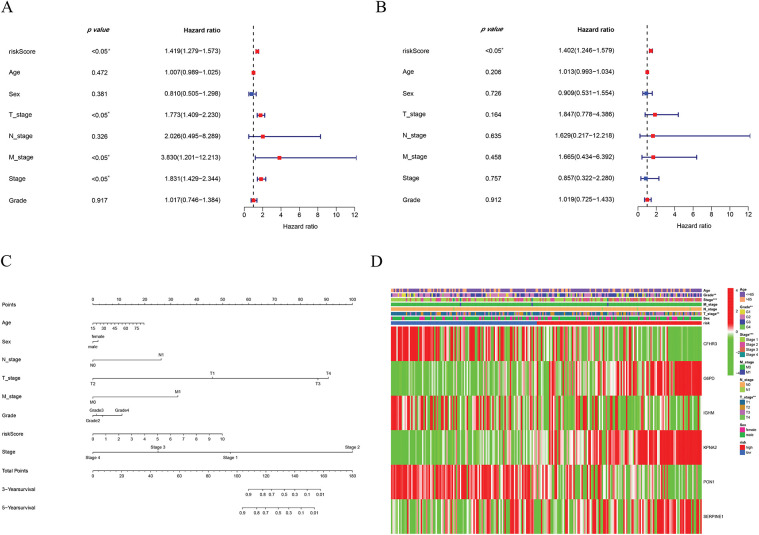

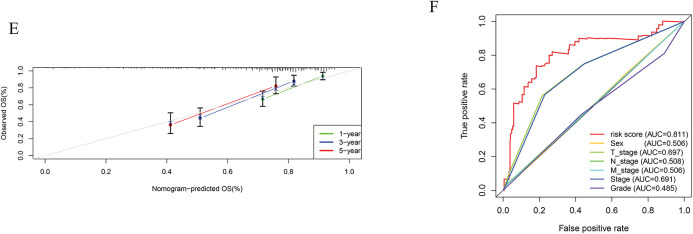
Establishment of the nomogram. (**A**) Univariate cox-regression. (**B**) Multivariate cox-regression analysis. (**C**) Prognostic nomogram. (**D**) Clinical features of high- and low-risk patients. (**E**) Calibration curve. (**F**) ROC curve of the predictive factors. *p < 0.05, **p < 0.01, ***p < 0.001

**Figure A1:**
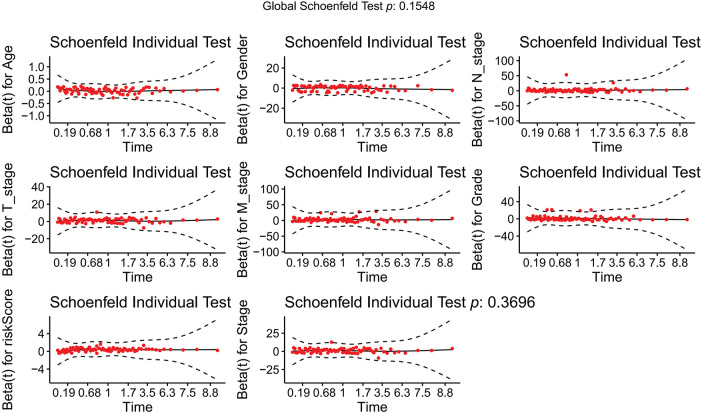
Schoenfeld goodness-of-fit test

Cox regression analyses, performed in both univariate and multivariate models, revealed that the risk score was significantly associated with overall prognosis in HCC patients (Fig. 5A,B). To further predict overall survival (OS), a comprehensive nomogram was developed by integrating clinical variables such as gender, age, tumor grade and stage, TNM status, along with the calculated risk score (Fig. 5C). The nomogram demonstrated robust predictive performance, with a concordance index of 0.748. Associations between clinical characteristics and the defined risk groups were visualized in a heatmap (Fig. 5D). The proportional hazards (PH) assumption was verified for all variables included in the nomogram using the Schoenfeld residuals test (Fig. A1, Table A2). Calibration plots demonstrated strong concordance between predicted and actual survival probabilities at 1, 3, and 5 years (Fig. 5E). Among all predictors, the risk score exhibited the highest area under the curve (AUC = 0.811) in ROC analysis, underscoring its superior prognostic accuracy (Fig. 5F).

### 3.4 Immune-Related Analyses

**Figure 6:**
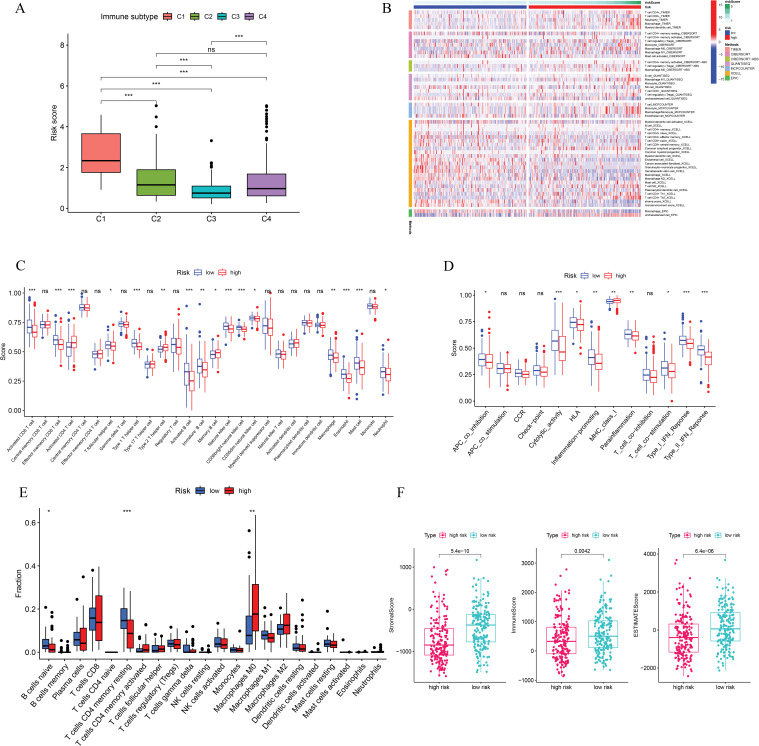
Immune cell infiltration analysis. (**A**) Immune subtypes of all HCC patients. (**B**) Immune cell infiltration status in high- and low-risk groups. (**C**) ssGSEA analysis based on the risk score. (**D**) Immune function analysis based on the risk score. (**E**) CIBERSORT analysis based on the risk score. (**F**) Immune, stromal, and ESTIMATE scores based on the risk score. *p < 0.05, **p < 0.01, ***p < 0.001; ns, no significant

**Figure A2:**
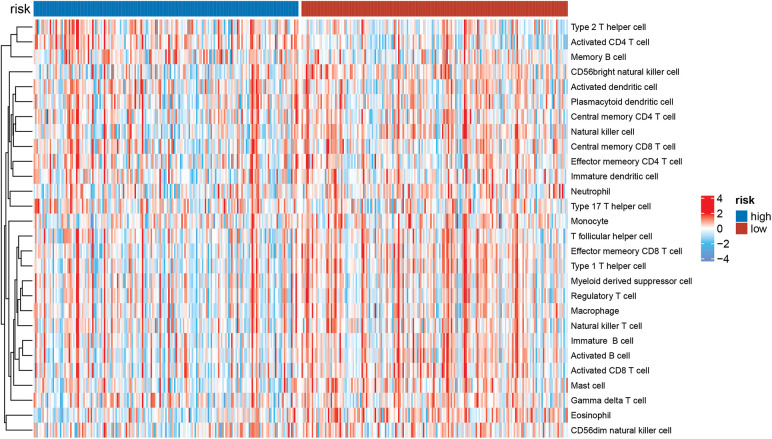
The immunologic landscape of HCC patients with ssGSEA method

**Figure A3:**
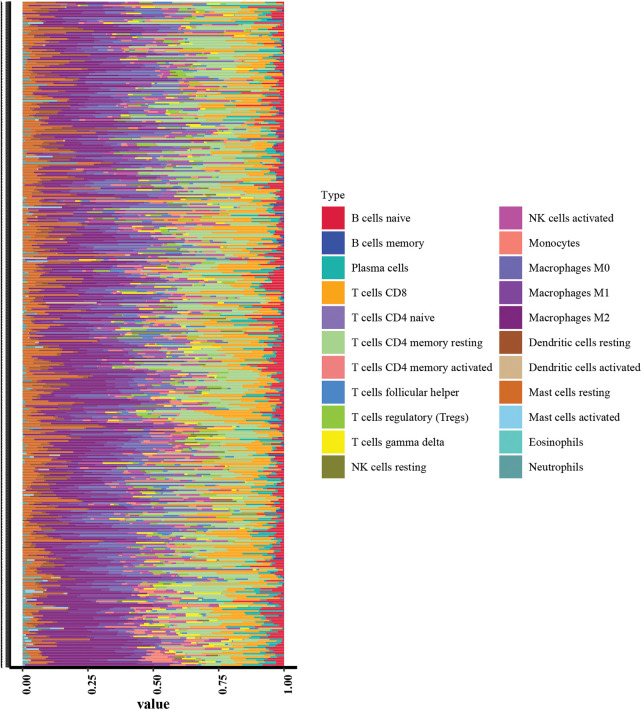
Immunologic landscape of HCC patients with CIBERSORT method

This study examined the immune subtypes of HCC patients across different risk score groups. High-risk patients were predominantly associated with the C1 (wound healing) subtype, while the C3 (inflammatory) subtype was more common among low-risk individuals (Fig. 6A). To investigate the association between immune cell infiltration and risk score further, multiple computational algorithms were employed. Analysis across multiple platforms consistently showed that individuals classified as low-risk had a markedly higher degree of immune cell infiltration (Fig. 6B). ssGSEA demonstrated elevated levels of various immune cells in this group, including activated and mature B lymphocytes, follicular helper T cells, effector memory and activated CD8^+^ T cells, Th1-type helper cells, natural killer (NK) cells, and macrophages (Fig. 6C). Conversely, the high-risk subgroup was characterized by increased activity in several immune-related processes, such as cytolytic function, pro-inflammatory signaling, T cell co-stimulatory pathways, and both type I and type II interferon responses (Fig. 6D). To gain further insight into the immune composition of individual tumors, the immune microenvironment was examined using both ssGSEA (Fig. A2) and the CIBERSORT deconvolution algorithm (Fig. A3). CIBERSORT analysis showed that low-risk patients had more naïve B cells and resting memory CD4^+^ T cells, while M0 macrophages were mainly enriched in the high-risk group (Fig. 6E). Furthermore, ESTIMATE results indicated that stromal, immune, and overall scores were significantly elevated in the low-risk group, suggesting a more active immune microenvironment (Fig. 6F).

**Figure 7:**
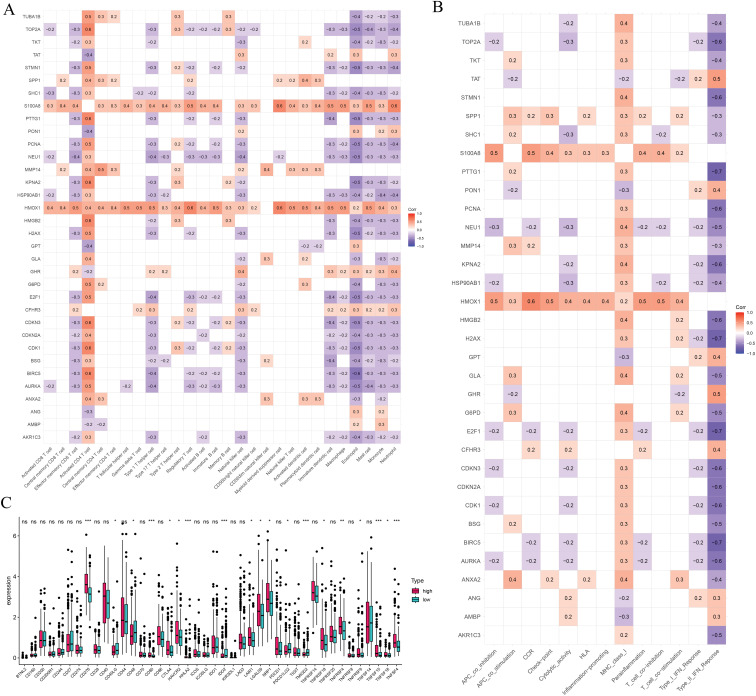
Immune-related analysis. (**A**) The correlation in prognostic ICD-related genes with immune cells. (**B**) The correlation in prognostic ICD-related genes with immune function pathways. (**C**) Immune checkpoints gene expression based on the risk score. *p < 0.05, **p < 0.01, ***p < 0.001; ns, no significant

We further explored the associations between prognostic ICD-related genes, immune cell infiltration, and immune functional pathways. CFHR3 expression showed a positive correlation with multiple immune cell types, such as γδ T cells, regulatory T cells (Tregs), Th1 helper T cells, NK cells, and CD8^+^ cytotoxic T lymphocytes. In contrast, G6PD expression was positively correlated with both activated and central memory CD4^+^ T cells, as well as with activated dendritic cells, but showed a negative association with NK cell abundance. KPNA2 was found to be upregulated in the presence of activated CD4^+^ T lymphocytes and Th2 cells, whereas its expression was negatively linked to Th1 cells, effector memory CD8^+^ T cells, and NK cells. In contrast, PON1 exhibited a strong positive correlation with NK cells and an inverse relationship with activated CD4^+^ T cells (Fig. 7A). Additionally, CFHR3, G6PD, KPNA2, and PON1 were each involved in multiple immune functional pathways (Fig. 7B). These findings indicate that the ICD-based risk score effectively reflects critical aspects of the immune microenvironment in hepatocellular carcinoma and may serve as a valuable biomarker for differentiating immune phenotypes.

**Figure 8:**
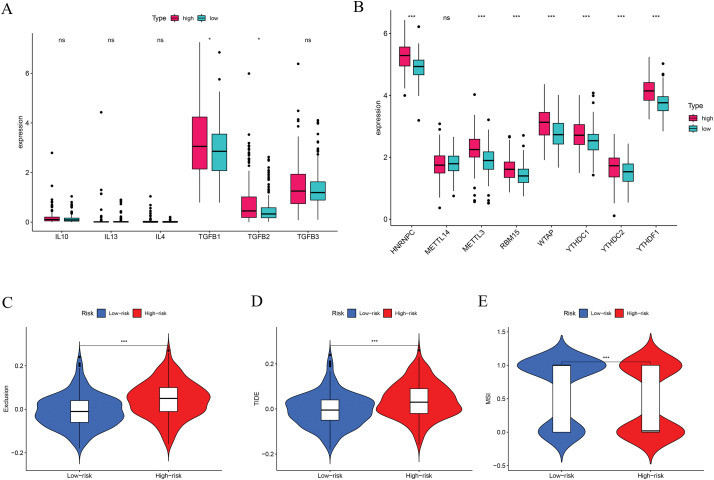
Immune-suppressive factors, m6A methylation, and immunotherapy response analysis. (**A**) Association between the immune-suppressive factors and risk score. (**B**) Association between the m6A methylation and risk score. (**C**–**E**) Immunotherapy response based on the risk score. *p < 0.05, ***p < 0.001; ns, no significant

We also assessed immune checkpoint expression, immunosuppressive factors, and m6A methylation regulators across different risk groups. Eighteen immune checkpoint genes showed differential expression when comparing the low- and high-risk groups (Fig. 7C). Importantly, the immunosuppressive cytokines TGFβ1 and TGFβ2 were significantly elevated in patients classified as high-risk (Fig. 8A). Furthermore, analysis of m6A-related genes indicated that HNRNPC, METTL3, RBM15, WTAP, YTHDC1, YTHDC2, and YTHDF1 exhibited notably higher expression levels in the high-risk cohort (Fig. 8B). Furthermore, the TIDE algorithm was utilized to estimate the potential responsiveness to immunotherapy. Patients classified as high-risk showed markedly elevated TIDE and Exclusion scores, accompanied by reduced microsatellite instability (MSI) levels (Fig. 8C–E), indicating a lower probability of favorable outcomes from immune checkpoint inhibitor treatment.

### 3.5 Drug Sensitivity Analysis

**Figure 9:**
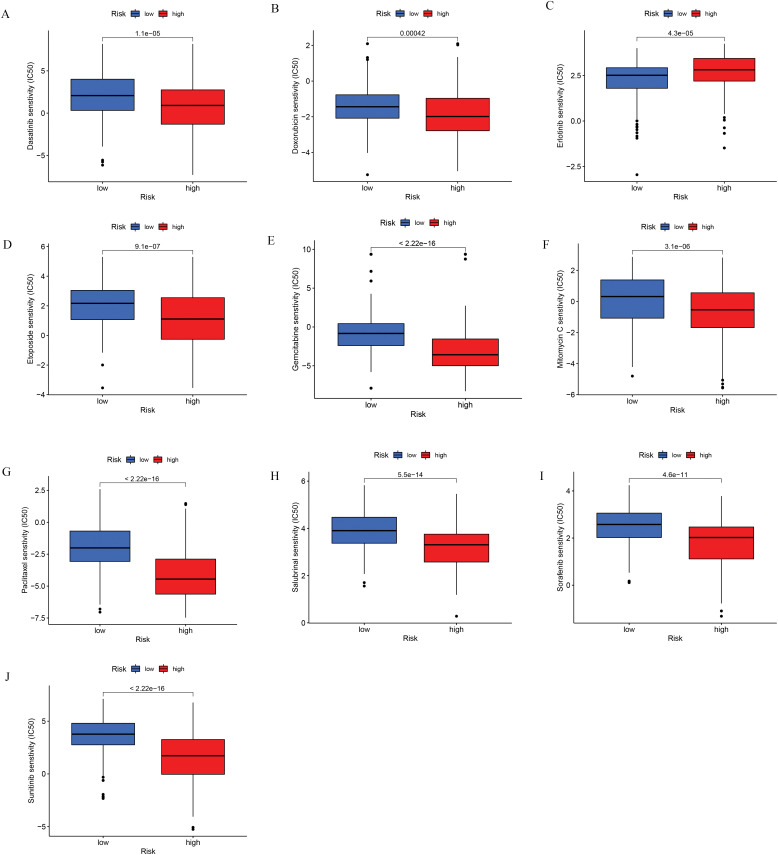
Chemotherapeutic sensitivity analysis. (**A**) Dasatinib; (**B**) Doxorubicin; (**C**) Erlotinib; (**D**) Etoposide; (**E**) Gemcitabine; (**F**) Mitomycin C; (**G**) Paclitaxel; (**H**) Salubrinal; (**I**) Sorafenib; (**J**) Sunitinib

Drug sensitivity analyses based on data from the Cancer Genome Project were conducted to compare responses between low- and high-risk patient groups. Findings revealed that individuals with higher risk scores exhibited greater sensitivity to several HCC treatments, such as doxorubicin, gemcitabine, mitomycin C, paclitaxel, etoposide, sorafenib, and sunitinib (Fig. 9). These findings imply that the developed risk model could function as a reliable biomarker to inform and optimize systemic treatment approaches for hepatocellular carcinoma. Additional comparisons involving other anticancer agents are presented in Fig. A3.

### 3.6 Functional Enrichment Analysis

**Figure A4:**
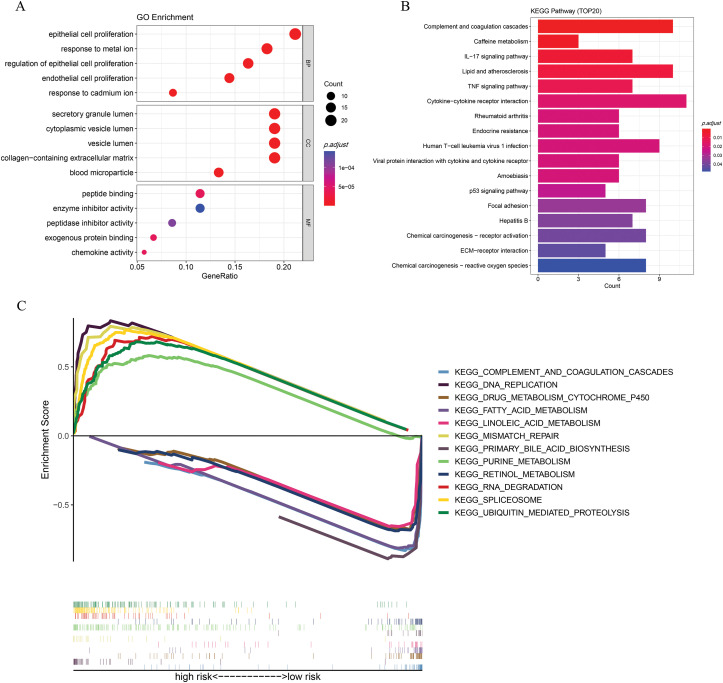
Functional enrichment analysis. (**A**) GO enrichment. (**B**) KEGG analysis. (**C**) GSEA analysis

GO enrichment analysis showed that differentially expressed ICD-associated genes were predominantly implicated in biological processes such as the proliferation of epithelial and endothelial cells, as well as responses to metal ion stimuli. Regarding cellular component (CC) categories, these genes were mainly localized to regions like the lumen of secretory granules and cytoplasmic vesicles. In terms of molecular function, they were significantly enriched in activities including exogenous protein interaction, peptide binding, and chemokine-related functions (Fig. A4A). Additionally, KEGG pathway analysis revealed associations with several signaling routes, notably the complement and coagulation cascades, caffeine metabolism, and IL-17 signaling (Fig. A4B). Additionally, GSEA showed that metabolism-related pathways were mainly enriched in the low-risk group, whereas the high-risk group was associated with nucleic acid-related processes, including RNA degradation, mismatch repair, and purine metabolism (Fig. A4C).

### 3.7 The Distribution and Expression Profiles of the Six ICD-Related Genes in a Single-Cell Dataset

**Figure 10:**
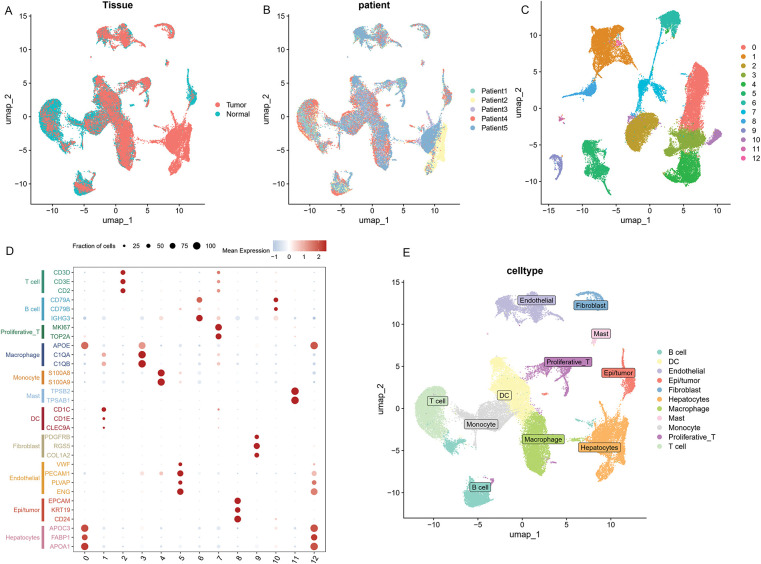

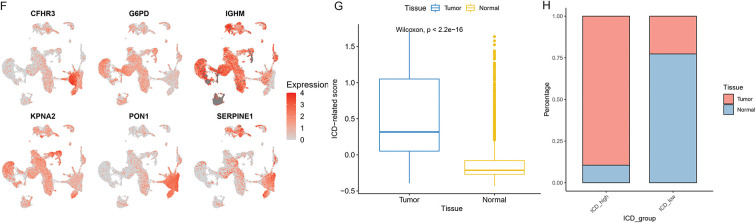
The distribution and expression profiles of the six ICD-related genes in a single-cell dataset. (**A**) The umap shows the tissue origin of 44,624 cells. (**B**) UMAP of single-cell clusters from patients with HCC, colored based on various patients. (**C**) Dimension reduction and cluster analysis. All cells were clustered into 13 clusters. (**D**) Dot plot showing representative marker genes for each cell cluster and type. (**E**) Eleven cell types are identified by marker genes. (**F**) UMAP plots that display the expression of six ICD-related genes across all cell types. (**G**) Differences of ICD-related scores of cells between different tissues. The statistical differences between groups were determined by the Wilcoxon rank test. (**H**) The proportion of cells derived from tumor and normal tissue in the ICD-high and ICD-low groups

As illustrated in Fig. 10A,B, ten samples—comprising tumor and adjacent normal tissues from five HCC patients—were included in the analysis. All single cells were subsequently clustered into 13 distinct populations (Fig. 10C), and cell types were annotated based on the gene expression profiles characteristic of each cluster (Fig. 10D,E). To illustrate the expression and spatial distribution of six key ICD-related genes in hepatocellular carcinoma, UMAP dimensionality reduction was applied (Fig. 10F). ICD scores for individual samples were calculated utilizing the AddModuleScore function, which integrates the expression data of these six signature genes. Notably, tumor tissues exhibited significantly elevated ICD scores compared to their paired normal counterparts (Fig. 10G). Moreover, elevated ICD scores were predominantly observed in tumors exhibiting more aggressive pathological features (Fig. 10H).

## 4 Discussion

HCC, acknowledged as one of the most fatal malignant tumors, is characterized by a poor prognosis. Despite recent advances in multidisciplinary therapy, the lack of monitoring and insufficient diagnostic accuracy results in a poor overall survival rate [2]. Therefore, achieving an early, accurate diagnosis, along with effective risk stratification, is invaluable for improving the prognosis of HCC. ICD is currently acknowledged as a key mechanism underlying the effectiveness of multiple cancer treatments, such as chemotherapy, radiotherapy, and photodynamic therapy. Hence, gaining insight into the mechanisms and clinical significance of ICD is crucial for the advancement of innovative cancer therapies.

Although recent developments in systemic therapies have contributed to slight gains in survival for HCC patients, the optimal treatment strategy has yet to be definitively established. An emerging strategy focuses on harnessing tumor cell death in conjunction with immune system activation to eradicate cancer cells. While some chemotherapy agents induce direct tumor cell death, this process is generally tolerogenic and does not engage the adaptive immune response, thereby limiting the long-term effectiveness of anti-tumor immunity [15]. Notably, certain drugs like anthracyclines, oxaliplatin, and doxorubicin have demonstrated the capacity not only to cause apoptosis directly in tumor cells but also to initiate ICD. As a controlled mode of cell death, ICD stimulates T cell-driven adaptive immunity, reactivates immune surveillance, and remodels the immunosuppressive tumor microenvironment, ultimately producing a sustained tumor-inhibitory effect [9,16]. ICD induction-based therapies may be a new direction for future immunotherapy for HCC. Therefore, further research focusing on the occurrence and mechanism of ICD is crucial to guide treatment and create more effective treatment options for HCC patients.

In this study, based on six ICD genes associated with prognosis, a risk model was developed to forecast the survival of HCC patients, and its reliability was validated. Our results suggested that patients at high risk exhibited poor survival compared with low-risk patients. The nomogram demonstrated that the risk model reliably predicts survival outcomes in patients with HCC. Multiple studies have investigated the role of ICD-related genes in hepatocellular carcinoma and have constructed prognostic models to predict patient outcomes [17,18]. In contrast to these studies, our study not only screened the genes most significantly associated with HCC patient prognosis and developed a corresponding prognostic model but also further explored the correlation between these genes and the immune status of HCC patients, such as immunophenotyping, immune cell infiltration status, immune function pathways, immune checkpoints, immunosuppressive factors, immunotherapy response, m6A methylation, and anti-tumor drug sensitivity. Furthermore, qRT-PCR and immunohistochemistry were performed to validate our results.

CFHR3 encodes complement factor H-related protein 3, a key regulator that inhibits activation of the complement system. Dysregulation of CFHR3 has been implicated in several diseases, including IgA nephropathy [19], atypical hemolytic-uremic syndrome [20], and biliary tract cancer [21]. As a key component of innate immunity, the complement system plays a dual role in tumorigenesis, by promoting and inhibiting cancer development [22]. Complement factor H-related proteins are associated with evasion of immune surveillance. Previous research has suggested CFHR3 as a novel prognostic biomarker for HCC, and its downregulation enhances tumor aggressiveness, correlating with higher T-stages, advanced clinical stages, and increased recurrence risk [23,24]. The activity of Glucose-6-phosphate dehydrogenase (G6PD) is elevated in numerous tumor types, and studies have demonstrated that suppressing G6PD function can slow the progression of HCC [25]. Furthermore, high expression of G6PD promotes HCC migration and invasion through epithelial-mesenchymal transition, leading to poor prognosis [26]. The IGHM gene is responsible for encoding the C region of the mu-heavy chain of IgM, which is crucial for determining the IgM isotype. Diseases associated with IGHM include agammaglobulinemia [27] and type 1 diabetes [28]. Its related immune pathways are the NFAT immune response and the lectin-induced complement pathways. IgM antibodies play a critical role in recognizing and clearing precancerous and cancerous lesions [29]. The IGHM gene is upregulated in breast cancer, where it notably influences the infiltration of CD20^+^ B cells and is linked to patient prognosis [30]. Similar outcomes were observed in ovarian cancer as well [31]. Karyopherin α2 (KPNA2) is commonly upregulated across multiple cancer types and serves a crucial function in nuclear transport. By modulating processes such as cell differentiation, proliferation, and apoptosis, KPNA2 facilitates tumor growth and progression. Elevated levels of KPNA2 are correlated with unfavorable clinical outcomes [32]. Overexpression of KPNA2 positively affects tumor differentiation, stage and vascular invasion, and can predict early recurrence and poor prognosis of HCC [33]. Paraoxonase 1 (PON1), predominantly found in the liver, functions as an antioxidant. Decreased serum PON1 activity in cancer patients has been associated with tumor stage and prognosis [34]. Serum PON1 level is also negatively associated with the degree of microvascular invasion and is a potential biomarker for HCC [35]. The SERPINE1 gene encodes serine protease inhibitor clade E member 1, which serves as a critical inhibitor of tissue plasminogen activator (tPA) and urokinase (uPA). Increased SERPINE1 expression has been detected in various cancers and is linked to pro-angiogenic activity, facilitation of tumor growth and migration, and suppression of apoptosis. Together, these roles promote tumor progression, invasiveness, and metastatic capability. Moreover, SERPINE1 has been identified as a prognostic indicator in gastric cancer, breast cancer, and various other malignancies [36,37].

Thorsson and colleagues introduced an immunogenomic framework that categorizes tumors into six distinct immune subtypes based on the characteristics of their tumor microenvironment (TME). These subtypes include wound healing (C1), IFN-γ dominant (C2), inflammatory (C3), lymphocyte-depleted (C4), immunologically quiet (C5), and TGF-β dominant (C6) [38], each reflecting unique immune profiles within the tumor milieu. Among these, the C3 subtype was associated with the most favorable prognosis, consistent with our findings showing that C3 patients had the lowest risk scores. CD4^+^ T cells play an essential role in initiating anti-tumor immune responses by activating CD8^+^ cytotoxic lymphocytes, the primary cells responsible for targeting and destroying tumor cells. Considerable research shows that CD4^+^ and CD8^+^ T cell levels are reduced in HCC tissues, while greater infiltration of these lymphocytes is linked to enhanced survival rates [39,40]. CD4^+^ T cells are able to differentiate into multiple subsets, including Th1 cells, Th2 cells, and follicular helper T (Tfh) cells. Tfh cells are essential for B cell activation within germinal centers and contribute to anti-tumor immunity through CD8^+^ T cell-dependent mechanisms. Reduced Tfh cell function has been linked to poor responses to anti-PD-1 immunotherapy [41]and contributes to the pathogenesis of HBV-related HCC [42]. Th1 and Th2 cells exhibit opposing roles in tumor biology, with Th1 cells acting as tumor suppressors and Th2 cells promoting tumor progression [43]. Th1 cells, through the secretion of IFN-γ, mediate anti-tumor effects by promoting apoptosis, inhibiting angiogenesis, enhancing tumor immunogenicity, and recruiting CD8^+^ T and NK cells [44]. In contrast, Th2 cells produce IL-4, a cytokine that promotes tumor cell growth and enhances metastatic potential. [45]. Clinical studies have shown that Th1-associated cytokines are predictive of better prognosis, while Th2 cytokines are linked to tumor invasion [46]. NK cells, though inherently capable of eliminating tumor cells, often exhibit functional exhaustion in HCC despite increased presence, ultimately contributing to immune evasion and disease progression [47]. Nevertheless, higher levels of NK cells within tumors have been linked to improved prognosis in HCC patients treated with sorafenib. [48].

Based on our results, high-risk patients present with decreased immune cell infiltration. The level of anti-tumor effector cells declined and the proportion of immunosuppressive cells increased. In combination with the immune function analysis, the activity of tumor suppressor pathways was downregulated. We hypothesized that high-risk patients would have impaired tumor-suppressing immune cells and immune pathways and an increased proportion of immune components that exert pro-tumor effects. Therefore, high-risk patients have a poor prognosis.

Immune checkpoints are inhibitory proteins found on effector lymphocytes that serve to prevent excessive immune responses. Among various immunotherapeutic strategies, the use of immune checkpoint inhibitors (ICIs) has gained the most traction due to their favorable efficacy across multiple tumor types. However, the available data suggest that ICIs have limited efficacy in HCC and there is still a need to optimize the current treatment strategy to achieve the best treatment responses. The TIDE analysis suggests that high-risk patients are more prone to immune escape and exhibit a diminished response to immunotherapy, aligning with our observation that this group displays an “immunologically cold” tumor microenvironment. Moreover, we found that several immune checkpoints, such as LAIR1, were upregulated in these patients. ICIs targeting these molecules have the potential to increase response rates, consequently boosting the overall effectiveness of immunotherapy. Chemotherapy is a valuable treatment option for HCC. We assessed the sensitivity of standard chemotherapeutic agents commonly employed in the treatment of HCC and found that patients classified as high-risk exhibited lower IC50 values for dasatinib, doxorubicin, gemcitabine, etoposide, mitomycin, paclitaxel, salubrinal, sorafenib, and sunitinib, indicating increased drug susceptibility in this group. Among these drugs, doxorubicin, paclitaxel, and salubrinal have been identified as ICD inducers. Previous studies have proposed that ICDs can trigger anti-tumor immune responses to potentiate the benefits of chemotherapy. For example, a previous study demonstrated that paclitaxel triggers ICD in ovarian cancer by activating cytotoxic mechanisms mediated via TLR4, IKK2, and SNARE proteins. This finding aligns with our GO analysis, which highlights the association between ICD and vesicular trafficking processes in cells [49]. In conclusion, our risk model can help elaborate on appropriate immunotherapy and chemotherapy strategies for specific patients and achieve personalized treatment goals.

However, several limitations should be acknowledged in this study. First, the study model was not validated by using external datasets. This is because the HCC datasets with clinical information available on the GEO platform do not include the IGHM gene. Similarly, the Japanese HCC dataset in the ICGC database also lacked IGHM expression data. While the ICGC France-HCC cohort does provide IGHM expression data, only six patients retained relevant clinical information after data cleaning, rendering it unsuitable for use as a validation dataset. Consequently, this risk model requires further validation using additional clinical data. Second, the immune features and drug sensitivity of patients with different risk scores have not been verified through cellular or animal experiments. Additionally, machine learning approaches such as Random Survival Forest Support Vector Machines, Random Forest, NNET Boruta, and K-Nearest Neighbors have become increasingly prevalent in recent years. While various modeling approaches have emerged, the fundamental goal remains the same: to identify hub genes and develop a prognostic model with good predictive performance that is also simple and applicable in clinical practice. One of the limitations of our study is that the method used for key risk gene selection and model construction lacks novelty. Finally, owing to the scarcity of relevant research, the biological role and underlying molecular mechanisms of IGHM in HCC remain insufficiently understood.

## 5 Conclusions

We constructed an ICD-related prognostic signature for HCC based on six ICD-associated risk genes: CFHR3, G6PD, IGHM, KPNA2, PON1, and SERPINE1. Single-cell sequencing and transcriptomic analyses showed that this signature effectively forecasts HCC patient prognosis and could provide important insights for treatment strategies.

## Data Availability

Both the clinical and the RNA-seq data (Genomic Data Commons (GDC), the Cancer Genome Atlas (TCGA), Liver Cancer (LIHC)) were available from the University of California, Santa Cruz (UCSC) Xena website (https://xena.ucsc.edu/, accessed on 18 June 2025). Clinical data set included age, sex, survival status, survival time, grade, and TNM stage. The R package “caret 6.0” was employed to randomly assign patients from the TCGA cohort into training and test sets in a 1:1 ratio. We retrieved the single-cell RNA-seq dataset GSE242889 from the Gene Expression Omnibus (GEO) database, which includes 10 samples derived from tumor and paired adjacent non-tumor tissues of five patients diagnosed with HCC. Protein-protein interactions (PPI) were analyzed using STRING (https://string-db.org, accessed on 18 June 2025). Immunohistochemistry (IHC) data were downloaded from the Human Protein Atlas (https://www.proteinatlas.org/, accessed on 18 June 2025).

## Appendix A

**Table A1:** The sequences of the primers used for the ICD-related genes

Gene	Forward	Reverse
G6PD	AGGCCACCCAGAGGAGAAG	CGATGATGCGGTTCCAGCCT
KPNA2	GACGTTCATTGACCAGGCGG	CATTACGCTCGCCTACGACT
CFHR3	GGGTGGTCACCAGCAGTACC	AGACCGTAGCCAGGATGGCA
PON1	TCTTGGGGATGGGACTGGCA	CCAGGACTGTTGGGGTTGAAGC
SERPINE1	AGGGTGTTTCAGCAGGTGGC	GGACCAGCTTCAGATCCCGC

**Table A2:** Schoenfeld individual test of nomogram

Variables	chisq	df	*p*-value
Age	1.113	1	0.291
Gender	0.789	1	0.375
N_stage	3.809	1	0.051
T_stage	3.373	3	0.338
M_stage	1.642	1	0.2
Grade	4.18	3	0.243
riskScore	2.67	1	0.102
Stage	3.147	3	0.37
GLOBAL	19.273	14	0.155
